# Metabolism of Zearalenone in the Rumen of Dairy Cows with and without Application of a Zearalenone-Degrading Enzyme

**DOI:** 10.3390/toxins13020084

**Published:** 2021-01-22

**Authors:** Christiane Gruber-Dorninger, Johannes Faas, Barbara Doupovec, Markus Aleschko, Christian Stoiber, Andreas Höbartner-Gußl, Karin Schöndorfer, Manuela Killinger, Qendrim Zebeli, Dian Schatzmayr

**Affiliations:** 1 BIOMIN Research Center, BIOMIN Holding GmbH, 3430 Tulln, Austria; johannes.faas@biomin.net (J.F.); barbara.doupovec@biomin.net (B.D.); markus.aleschko@biomin.net (M.A.); christian.stoiber@biomin.net (C.S.); andreas.hoebartner-gussl@biomin.net (A.H.-G.); karin.schoendorfer@biomin.net (K.S.); manuela.killinger@biomin.net (M.K.); dian.schatzmayr@biomin.net (D.S.); 2 Institute of Animal Nutrition and Functional Plant Compounds, Department for Farm Animals and Veterinary Public Health, University of Veterinary Medicine Vienna, 1210 Vienna, Austria; Qendrim.Zebeli@vetmeduni.ac.at

**Keywords:** mycotoxin, zearalenone, rumen, metabolism, degradation, hydrolase, feed additive

## Abstract

The mycotoxin zearalenone (ZEN) is a frequent contaminant of animal feed and is well known for its estrogenic effects in animals. Cattle are considered less sensitive to ZEN than pigs. However, ZEN has previously been shown to be converted to the highly estrogenic metabolite α-zearalenol (α-ZEL) in rumen fluid in vitro. Here, we investigate the metabolism of ZEN in the reticulorumen of dairy cows. To this end, rumen-fistulated non-lactating Holstein Friesian cows (*n* = 4) received a one-time oral dose of ZEN (5 mg ZEN in 500 g concentrate feed) and the concentrations of ZEN and ZEN metabolites were measured in free rumen liquid from three reticulorumen locations (reticulum, ventral sac and dorsal mat layer) during a 34-h period. In all three locations, α-ZEL was the predominant ZEN metabolite and β-zearalenol (β-ZEL) was detected in lower concentrations. ZEN, α-ZEL and β-ZEL were eliminated from the ventral sac and reticulum within 34 h, yet low concentrations of ZEN and α-ZEL were still detected in the dorsal mat 34 h after ZEN administration. In a second step, we investigated the efficacy of the enzyme zearalenone hydrolase ZenA (EC 3.1.1.-, commercial name ZEN*zyme*^®^, BIOMIN Holding GmbH, Getzersdorf, Austria) to degrade ZEN to the non-estrogenic metabolite hydrolyzed zearalenone (HZEN) in the reticulorumen in vitro and in vivo. ZenA showed a high ZEN-degrading activity in rumen fluid in vitro. When ZenA was added to ZEN-contaminated concentrate fed to rumen-fistulated cows (*n* = 4), concentrations of ZEN, α-ZEL and β-ZEL were significantly reduced in all three reticulorumen compartments compared to administration of ZEN-contaminated concentrate without ZenA. Upon ZenA administration, degradation products HZEN and decarboxylated HZEN were detected in the reticulorumen. In conclusion, endogenous metabolization of ZEN in the reticulorumen increases its estrogenic potency due to the formation of α-ZEL. Our results suggest that application of zearalenone hydrolase ZenA as a feed additive may be a promising strategy to counteract estrogenic effects of ZEN in cattle.

## 1. Introduction

Zearalenone (ZEN) is one of the most frequently detected mycotoxins in cereals and animal feed worldwide [1,2]. ZEN binds to estrogen receptors and exerts estrogenic effects in different animal species [3,4]. While pigs are particularly sensitive to ZEN, estrogenic effects have also been reported for ruminants. Hyperestrogenism was reported for dairy cows [5,6] and ewes [7] exposed to relatively high ZEN doses. Furthermore, a study in breeding cows suggested that a low dietary ZEN level (0.1 mg/kg in straw) did not affect fertility, but affected anti-Müllerian hormone levels in blood [8].

Rumen microbiota can degrade certain mycotoxins (e.g., deoxynivalenol], [9,10]) to less toxic substances and thus exert a protective function. However, in case of ZEN, microbiota-mediated metabolization in the rumen may not result in detoxification. Previous studies investigated the fate of ZEN in rumen fluid in vitro [11,12,13] and the flow of ZEN metabolites out of the rumen into the duodenum in cows that received ZEN-contaminated feed [14,15,16]. In either case, ZEN was found to be converted to α-zearalenol (α-ZEL) and β-zearalenol (β-ZEL). Same as their parent compound, α-ZEL and β-ZEL are estrogenic. However, α-ZEL is 60 times as potent as ZEN, whereas β-ZEL is only 0.2 times as potent as ZEN [17]. In vitro, ZEN was mainly converted to α-ZEL and to a lesser extent to β-ZEL in rumen fluid [11,12,13]. In duodenal digesta, more β-ZEL than α-ZEL was detected [14,15,16]. However, due to the higher estrogenicity of α-ZEL compared to ZEN, the combined estrogenic potency of all ZEN metabolites present in duodenal digesta was increased relative to the potency of ZEN ingested by the cow. These studies suggest that ZEN metabolization in the rumen results in increased estrogenic potency. However, to date, ZEN kinetics have not been investigated directly in the rumen. Additionally, it is unclear whether ZEN kinetics vary between rumen compartments. This would be plausible, as digesta in different locations differ physiologically and microbiologically [18,19].

**Figure 1:**
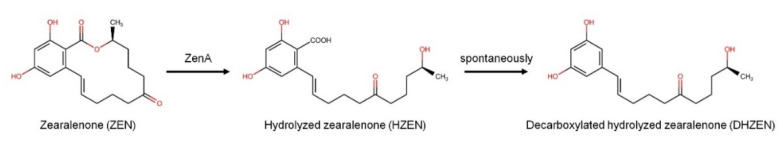
Enzymatic degradation of zearalenone by zearalenone hydrolase ZenA. Modified from [21,22].

Enzymes added to animal feed may enable ZEN detoxification in the rumen and prevent the formation of estrogenic metabolites. The enzyme zearalenone hydrolase ZenA (ZEN*zyme*^®^, BIOMIN Holding GmbH, Getzersdorf, Austria) converts ZEN to hydrolyzed ZEN (HZEN) by hydrolyzing the ester bond of its lactone ring (Figure 1). HZEN spontaneously converts to decarboxylated HZEN (DHZEN; Figure 1). Enzymatic conversion of ZEN to HZEN enables a strong reduction in estrogenic potency. HZEN and DHZEN did not elicit an estrogenic response in an MCF-7 cell proliferation assay [20,21] or an estrogen-sensitive yeast bioassay [21]. Moreover, in contrast to ZEN, HZEN and DHZEN did not affect reproductive tract morphology or expression of ZEN-responsive microRNAs in pigs [21]. Therefore, ZenA applied as a feed additive may reduce the estrogenic potency of dietary ZEN in the rumen.

In this study, we aimed to study the kinetics of ZEN degradation in various reticulorumen locations, and the efficacy of ZenA to favorably alter these kinetics. We hypothesized that ZEN is converted to α-ZEL and β-ZEL in the rumen and that ZenA converts ZEN to HZEN thereby preventing α-ZEL and β-ZEL formation. First, we assessed the efficacy of ZenA to degrade ZEN in a simulated rumen environment in vitro. Second, we performed a feeding trial in rumen-fistulated non-lactating dairy cows to study ZEN degradation kinetics in presence and absence of ZenA. To our knowledge, this is the first published study that investigates ZEN kinetics and the efficacy of a ZEN-degrading enzyme directly in the rumen.

## 2. Results

### 2.1. Degradation of ZEN by ZenA in a Simulated Rumen Environment In Vitro

**Figure 2:**
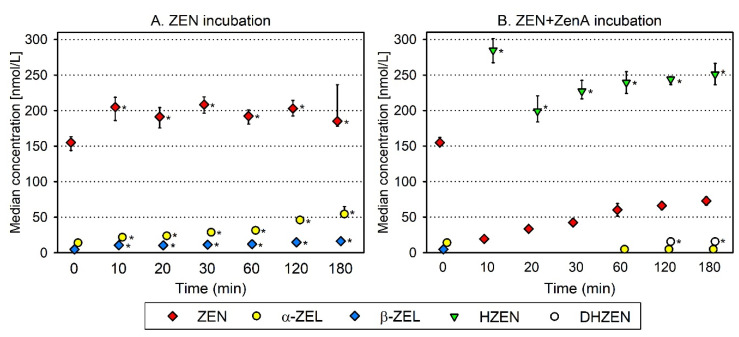
Metabolization and enzymatic degradation of zearalenone in a simulated rumen environment. (**A**) shows concentrations of zearalenone (ZEN) and its metabolites α-zearalenol (α-ZEL), β-zearalenol (β-ZEL), hydrolyzed ZEN (HZEN) and decarboxylated HZEN (DHZEN) in reactors incubated with ZEN. (**B**) shows concentrations of ZEN and its metabolites in reactors incubated with ZEN and ZenA. Symbols (red diamond—ZEN; yellow circle—α-ZEL; blue diamond—β-ZEL; green triangle—HZEN; white circle—DHZEN) indicate median of four replicates and error bars indicate interquartile range. If a compound was detectable but below the limit of quantification (LOQ), the concentration in the respective sample was assumed to be LOQ/2 for calculation of median and interquartile range. If a compound was below the limit of detection in all four replicates at a given time point, no symbol is depicted. HZEN and DHZEN were not detected in reactors incubated with ZEN (**A**) at any time point. Asterisks indicate a significantly higher concentration (*p* < 0.05) in treatment ZEN compared to ZEN+ZenA or in treatment ZEN+ZenA compared to ZEN for the respective sampling location and time point.

We investigated the efficacy of ZenA to degrade ZEN in a batch fermentation system simulating a rumen environment. In reactors incubated with ZEN and without ZenA, estrogenic ZEN metabolites α-ZEL and β-ZEL were formed and showed a slight increase during 3 h of incubation (Figure 2A). In reactors incubated with ZEN and ZenA (Figure 2B), concentrations of ZEN, α-ZEL and β-ZEL were significantly lower (*p* < 0.05) compared to reactors incubated without ZenA (Figure 2A) from 10 min to 3 h after the addition of the enzyme. At the same time, the non-estrogenic degradation product HZEN was detected at a high concentration (Figure 2B). DHZEN was first detected 2 h after the start of the incubation (Figure 2B).

### 2.2. Metabolism of ZEN in the Reticulorumen of Dairy Cows

**Figure 3:**
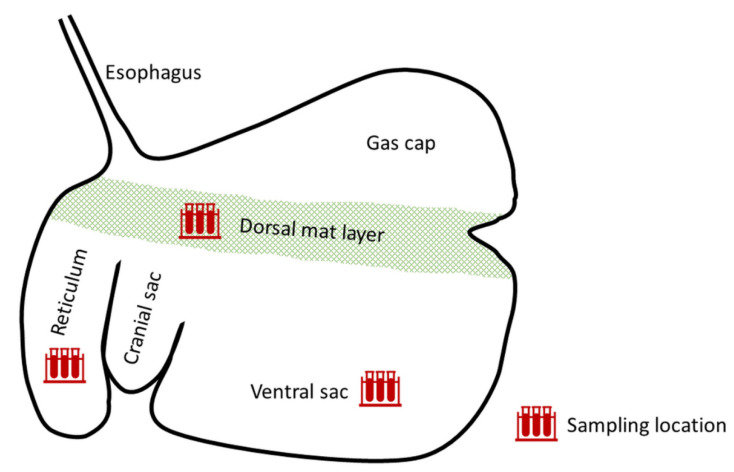
Scheme of the reticulorumen of a dairy cow. Red symbols in the shape of reaction tubes indicate sampling locations.

**Figure 4:**
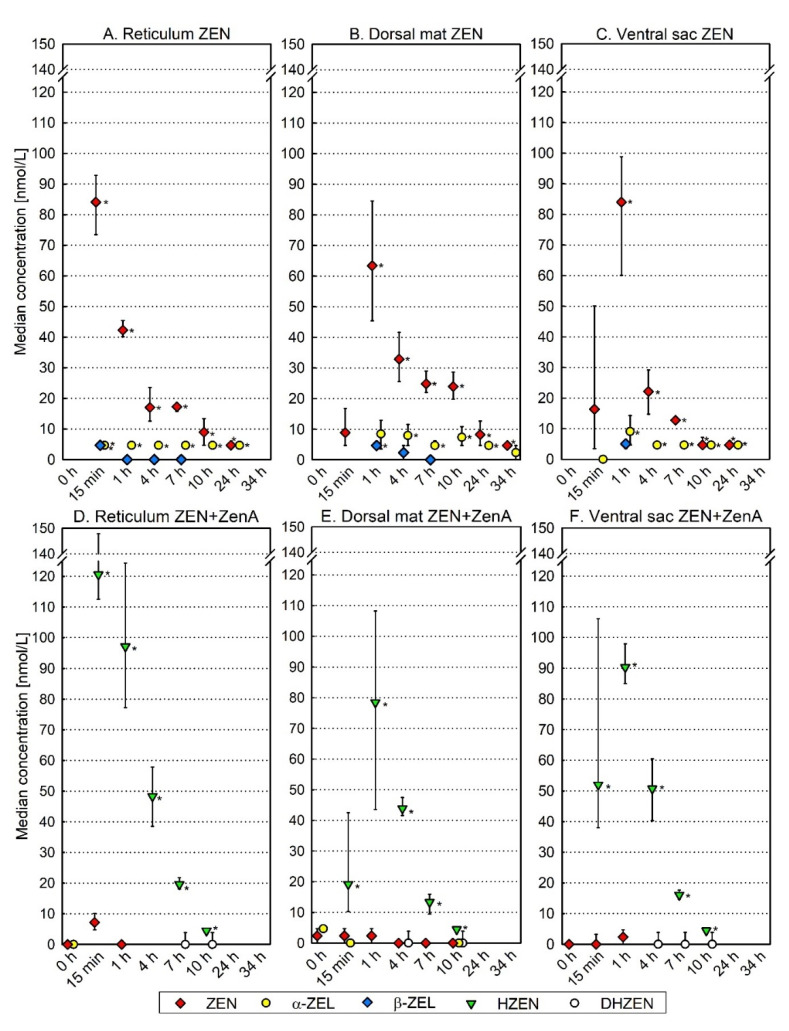
Metabolization and enzymatic degradation of zearalenone in the reticulorumen of dairy cows. Subfigures in the top row show concentrations of ZEN and its metabolites in reticulum (**A**), dorsal mat layer (**B**) and ventral sac (**C**) after feeding ZEN-contaminated concentrate (“ZEN”; experimental days 1 and 2). Subfigures in the bottom row show concentrations of ZEN and its metabolites in reticulum (**D**), dorsal mat layer (**E**) and ventral sac (**F**) after feeding ZEN-contaminated concentrate supplemented with ZenA (“ZEN+ZenA”; experimental days 3 and 4). Symbols (red diamond—ZEN; yellow circle—α-ZEL; blue diamond—β-ZEL; green triangle—HZEN; white circle—DHZEN) indicate median of four replicates and error bars indicate interquartile range. If a compound was detectable but below the limit of quantification (LOQ), the concentration in the respective sample was assumed to be LOQ/2 for calculation of median and interquartile range. If a compound was below the limit of detection in all four replicates at a given location and time point, no symbol is depicted. HZEN and DHZEN were not detected in any reticulorumen location after ZEN treatment (**A**–**C**). β-ZEL was not detected in any reticulorumen location after ZEN+ZenA treatment (**D**–**F**). α-ZEL was not detected in ventral sac after ZEN+ZenA treatment (**F**). Asterisks indicate a significantly higher concentration (*p* < 0.05) in treatment ZEN compared to ZEN+ZenA or in treatment ZEN+ZenA compared to ZEN for the respective sampling location and time point. Abbreviations: ZEN-zearalenone; α-ZEL-α-zearalenol; β-ZEL-β-zearalenol; HZEN-hydrolyzed ZEN; DHZEN-decarboxylated HZEN.

We investigated the metabolism of ZEN in the reticulorumen of fistulated dairy cows. To this end, cows received a one-time bolus of ZEN-contaminated concentrate feed (“ZEN” treatment) and concentrations of ZEN and its metabolites were measured in rumen fluid obtained from reticulum, dorsal mat layer and ventral sac (Figure 3) during a 34-h period. After administration of concentrate feed, ZEN was detected in rumen fluid obtained from reticulum, dorsal mat layer and ventral sac from the first sampling time point (15 min) onwards (Figure 4A–C). In reticulum (Figure 4A) and ventral sac (Figure 4C), ZEN was detected up until 24 h after concentrate feeding, while in the dorsal mat layer (Figure 4B), low concentrations of ZEN were still detected 34 h after concentrate feeding. In the reticulum, the ZEN concentration peaked 15 min post-administration (Figure 4A). In the dorsal mat layer (Figure 4B) and in the ventral sac (Figure 4C), the ZEN concentration peaked 1 h post-administration. α-ZEL appeared for the first time 15 min after concentrate feeding in reticulum and ventral sac and was detected up until 24 h after feeding in these compartments (Figure 4A,C). In the dorsal mat layer, α-ZEL was first detected 1 h after concentrate feeding and it was still detected at low concentrations 34 h after concentrate feeding (Figure 4B). β-ZEL was detected at low concentrations in the beginning of the sampling period in all three compartments (after 15 min to 7 h in the reticulum, after 1 to 7 h in the dorsal mat layer and after 1 h in the ventral sac; Figure 4A–C). α-ZEL was the main ZEN metabolite in the reticulorumen at every sampling time point (Table 1; Figure 4A–C). It was consistently detected at a higher concentration than β-ZEL and its concentration was stable up until 24 h after concentrate feeding, while the concentration of ZEN steadily declined (Table 1; Figure 4A–C).

**Figure 5:**
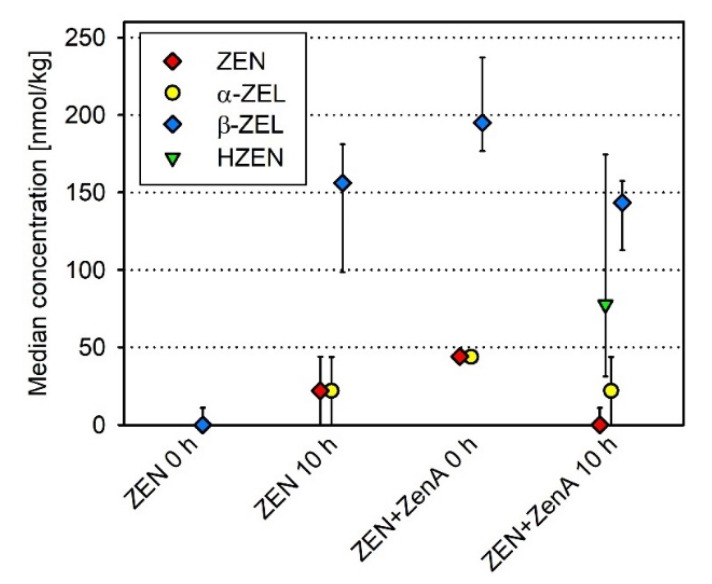
Concentrations of zearalenone and its metabolites in feces of dairy cows after feeding ZEN-contaminated concentrate (“ZEN”; experimental day 1) and after feeding ZEN-contaminated concentrate supplemented with ZenA (“ZEN+ZenA”; experimental day 3). Symbols (red diamond—ZEN; yellow circle—α-ZEL; blue diamond—β-ZEL; green triangle—HZEN) indicate median of four replicates and error bars indicate interquartile range. If a compound was detectable but below the limit of quantification (LOQ), the concentration in the respective sample was assumed to be LOQ/2 for calculation of median and interquartile range. If a compound was below the limit of detection in all four replicates at a given time point, no symbol is depicted. DHZEN was not detected in any sample. Abbreviations: ZEN—zearalenone; α-ZEL—α-zearalenol; β-ZEL—β-zearalenol; HZEN—hydrolyzed ZEN; DHZEN—decarboxylated HZEN.

Concentrations of ZEN and ZEN metabolites in feces were analyzed before concentrate feeding (0 h) and 10 h after concentrate feeding. ZEN, α-ZEL and β-ZEL were detected in feces 10 h after concentrate feeding (Figure 5). In contrast to what was observed in rumen fluid, β-ZEL was detected at a higher concentration than α-ZEL and ZEN in feces (Figure 5; Table 1).

### 2.3. Effect of ZenA on the Metabolism of ZEN in the Reticulorumen of Dairy Cows

Following the investigation of ZEN metabolism in the reticulorumen, we evaluated the efficacy of ZenA applied as a feed additive to detoxify ZEN. After administration of ZEN-contaminated concentrate supplemented with ZenA (“ZEN+ZenA” treatment), the concentrations of ZEN and α-ZEL were significantly lower (*p* < 0.05) and the concentration of HZEN was significantly higher (*p* < 0.05) in all three reticulorumen locations at most sampling time points compared to the ZEN treatment (Figure 4). β-ZEL was not detected at any sampling time point (Figure 4D–F). HZEN was detected as main ZEN metabolite in all three reticulorumen compartments (Figure 4D–F; Table 1) and it showed a similar trend as observed for ZEN in the ZEN treatment (Figure 4A–C), with a peak 15 min to 1 h after ingestion of feed and a steady decline thereafter. It was not detected anymore 24 h after concentrate feeding (Figure 4D–F). During the entire sampling period, ZEN and α-ZEL were detected at low concentrations or were not detected (Figure 4D–F). Low concentrations of DHZEN were detected from 4 h to 10 h after concentrate feeding (Figure 4D–F).

In feces, significant concentrations of ZEN, α-ZEL and β-ZEL were already detected before concentrate feeding (0 h; Figure 5). These concentrations were similar or higher compared to the 10 h sampling time point of the ZEN treatment (Figure 5). The ZEN+ZenA treatment was performed 48 h after the ZEN treatment, using the same animals (see Section 5.2.1). The washout period between treatments was sufficient for the removal of ZEN and its metabolites from the reticulorumen (Figure 4), but evidently not from the entire digestive tract. Therefore, a comparison of ZEN and ZEN metabolite concentrations in feces between the ZEN treatment and the ZEN+ZenA treatment using statistical analysis is not meaningful. Nevertheless, the appearance of HZEN in feces 10 h after feeding of ZEN-contaminated concentrate supplemented with ZenA supports degradation of ZEN by ZenA in the digestive tract.

## 3. Discussion

### 3.1. Degradation of ZEN by ZenA in a Simulated Rumen Environment In Vitro

Enzymatic activity may be affected by a multitude of factors. Both for animal welfare and for economic reasons, it is advisable to carefully assess the activity of an enzyme intended to be applied as a feed additive under simulated digestive conditions in vitro prior to testing its efficacy in a feeding trial. In a previous publication, ZenA was shown to remove ZEN and prevent the formation of α-ZEL in an in vitro rumen simulation experiment [23]. However, degradation products of ZEN were not analyzed in this previous study. Here, we found that upon addition of ZenA to reactors simulating a rumen environment, most of the ZEN present in the reactors was converted to HZEN within the first 10 min of incubation, indicating a high enzymatic activity (Figure 2). HZEN partly decarboxylated to DHZEN during 2 h of incubation (Figure 2). Low concentrations of residual ZEN were detected throughout the incubation period. Degradation of residual ZEN could have been prevented due to inaccessibility to the enzyme. By contrast, in the in vivo situation, upon ZenA administration only very low concentrations of ZEN were detected (Figure 4D–F), indicating a good accessibility of ZEN to the enzyme. While concentrations of α-ZEL and β-ZEL steadily increased within 3 h in reactors incubated without ZenA, α-ZEL and β-ZEL concentrations were low or below limit of detection (LOD) in reactors incubated with ZenA (Figure 2). Significantly lower concentrations (*p* < 0.05) of α-ZEL and β-ZEL in ZenA-treated reactors indicate that rapid removal of ZEN by enzymatic degradation prevented formation of these estrogenic metabolites. We conclude that ZenA rapidly degraded ZEN to HZEN in a simulated rumen environment and HZEN decarboxylated to DHZEN. Furthermore, ZenA reduced the concentrations of estrogenic metabolites α-ZEL and β-ZEL.

### 3.2. Metabolism of ZEN in the Reticulorumen of Dairy Cows

In the reticulorumen, digesta is stratified in various layers [24,25,26] and recent data suggest dramatic differences in the microbiome of these layers and locations [18]. Thus, for a thorough investigation of ruminal ZEN degradation kinetics, we chose to sample rumen fluid at multiple locations in the reticulorumen, namely in the reticulum, dorsal mat layer and ventral sac (Figure 3). After feeding a single dose of ZEN-contaminated concentrate, the ZEN concentration rose quickly in rumen fluid from every sampling location (Figure 4A–C). The ZEN concentration peaked 15 min after concentrate feeding in the reticulum (Figure 4A) and 1 h after concentrate feeding in the dorsal mat layer (Figure 4B) and the ventral sac (Figure 4C). An earlier peak of ZEN in the reticulum compared to the other locations is to be expected, as, following ingestion, concentrate particles reach the reticulum first and are then passed on to the dorsal mat layer. The ventral sac contains extensively digested particles and concentrate particles reach this location after being released from the mat layer [24]. ZEN was eliminated from reticulum (Figure 4A) and ventral sac (Figure 4C) within 34 h after concentrate feeding. However, in the dorsal mat layer, ZEN was still detected at this sampling time point (Figure 4B). Moreover, traces of ZEN and α-ZEL were still detected in the dorsal mat layer at time point 0 h of the ZEN+ZenA treatment, i.e., 48 h after administration of ZEN-contaminated concentrate (Figure 4E). Consequently, ZEN-contaminated concentrate particles may remain entrapped in the mat layer for longer periods of time. Additionally, the microbiota of the dorsal mat could have been less active in the conversion of ZEN than the microbiota present in other locations. Typically, the pH of the dorsal mat is 0.5 units lower than that of the ventral rumen and reticulum [19]. A lower pH might negatively affect the metabolic activity of the microbiota.

Estrogenic ZEN metabolites α-ZEL and β-ZEL were first detected 15 min–1 h after concentrate feeding in reticulum, dorsal mat layer and ventral sac (Figure 4A–C). Appearance of these metabolites coincided with a peak in ZEN concentration, indicating a rapid onset of ZEN metabolization by the rumen microbiota. Interestingly, α-ZEL was the main metabolite of ZEN in every sampling location (Figure 4A–C, Table 1), and it co-occurred with ZEN up until 24 h after concentrate feeding in reticulum and ventral sac and up until 34 h after concentrate feeding in the dorsal mat layer. Given that α-ZEL is 60 times as estrogenic as ZEN [17], these results confirm that ZEN metabolization in the rumen results in increased estrogenic potency.

A higher concentration of α-ZEL than β-ZEL in the reticulorumen following ingestion of ZEN is in contrast to previous studies that detected β-ZEL at a higher proportion than α-ZEL in duodenal digesta [14,15,16] and in feces [14,27] of cows. In accordance with previous reports, β-ZEL was the main ZEN metabolite in feces in this study (Figure 5, Table 1). One explanation for these different proportions of α-ZEL and β-ZEL detected in reticulorumen and duodenum/feces may be a possible metabolization of ZEN predominantly to β-ZEL in the abomasum or the omasum. Alternatively, the higher proportion of β-ZEL compared to α-ZEL in duodenal digesta and feces may be due to metabolization of ZEN in the liver and biliary excretion. Indeed, β-ZEL has been reported to be more abundant than α-ZEL in the liver [28] and in bile [29,30,31] of ZEN-fed cattle. Furthermore, β-ZEL was the predominant metabolite of ZEN formed in hepatic microsomes from cattle [32]. Differences in proportions of α-ZEL and β-ZEL in the reticulorumen in this experiment compared to duodenal digesta in previous experiments may also be due to physiological or nutritional differences such as differences in the composition of the microbial community in the rumen or differences in feed intake, the latter of which has already been shown to affect ruminal ZEN metabolism [14,16,33].

### 3.3. ZEN-Degrading Enzyme ZenA as a New Strategy for Mycotoxin Inactivation in Feed

When cows received ZEN-contaminated concentrate supplemented with ZenA, ZEN and α-ZEL were only detected at low levels or were below the LOD in all three sampling locations of the reticulorumen throughout the 34-h sampling period (Figure 4D–F). Statistical analysis confirmed that concentrations of ZEN and α-ZEL were significantly lower (*p* < 0.05) after administration of ZEN-contaminated concentrate supplemented with ZenA (Figure 4D–F) than after administration of ZEN-contaminated concentrate without ZenA (Figure 4A–C). Consequently, ZenA readily degraded ZEN to HZEN in the reticulorumen, thereby counteracting the metabolization of ZEN to α-ZEL by rumen microbiota. As intended, application of ZenA as a feed additive strongly decreased the estrogenic potency of ZEN-contaminated feed.

Different approaches for the inactivation of mycotoxins in feedstuffs have been evaluated. Most mycotoxins show a high degree of chemical resistance and as chemical treatments are expensive and detrimental to the feed’s nutritional quality, they are mostly inadequate for eliminating mycotoxins [34]. Physical treatments (such as washing, sorting, dehulling, etc.) can be quite effective to reduce the level of mycotoxins in agricultural commodities, yet fail to eliminate them entirely [35]. The application of feed additives that prevent the absorption of mycotoxins from the gastrointestinal tract was shown to be well suited for the removal of certain mycotoxins. Adsorbents such as bentonite meant to bind mycotoxins, prevent their absorption and facilitate their excretion via feces have been proven effective for removing aflatoxins, but they adsorb ZEN less effectively [36,37]. By modifying the surface structure of clays, the binding capacity of ZEN can be improved [36,38], but the safety profile of these modified clays is unclear [39]. Biological transformation of mycotoxins by microorganisms or enzymes was recently recognized as a specific and effective approach for mycotoxin inactivation [40,41]. A fumonisin degrading enzyme, fumonisin esterase FumD (FUM*zyme*^®^, BIOMIN Holding GmbH, Getzersdorf, Austria), was already successfully applied as a fumonisin-deactivating feed additive in different animal species [42,43,44]. Several ZEN-degrading enzymes have been discovered and characterized recently [45,46,47]. Previously explored ideas for practical applications of ZEN-degrading enzymes included expression in transgenic plants [48,49] and application during the refining of corn oil [50]. This is to the best of our knowledge the first report on the successful use of a ZEN-degrading enzyme as a feed additive to detoxify ZEN in the gastrointestinal tract. We demonstrated the efficacy of ZenA to degrade ZEN in the rumen of dairy cows and these results likely translate to other ruminant species. Future work will address the efficacy of the enzyme in other animal species, and the safety of this approach for animals, consumers, workers and environment.

## 4. Conclusions

We found that the natural metabolization of ZEN in the rumen of dairy cows increased its estrogenic potency due to the formation of the highly estrogenic metabolite α-ZEL. Application of the enzyme ZenA (ZEN*zyme*^®^, BIOMIN Holding GmbH, Getzersdorf, Austria) as a feed additive facilitated the degradation of ZEN to non-estrogenic metabolites HZEN and DHZEN and prevented the formation of α-ZEL in the rumen. Therefore, application of ZenA as a feed additive may be a promising approach for detoxification of ZEN-contaminated feed.

## 5. Materials and Methods

### 5.1. In Vitro Rumen Fermentation Experiment

Rumen fluid from 3 bulls was obtained from a local slaughterhouse and pooled. An in vitro rumen batch fermentation system was established in 1 L Pyrex glass bottles (*n* = 8). To this end, a 10 g aliquot of a dairy cow total mixed ration (43.56% chopped grass hay, 35.65% ground corn grain, 15.05% shredded soy bean, 2.97% ground wheat grain, 1.35% limestone, 0.48% Ca_3_(PO_4_)_2_, 0.5% NaHCO_3_, 0.32% NaCl, 0.12% MgO) and 280 mg ZEN-containing culture material of *Fusarium graminearum* (resulting in a final ZEN concentration of 0.314 µM) were added to each reactor bottle. Subsequently, 1 L of a mixture containing 50% rumen fluid, 30% reverse osmosis (RO) water (produced using the the arium^®^ 61316 reverse osmosis system from Sartorius Biotech GmbH, Göttingen, Germany) and 20% synthetic saliva (0.06 g/L MgCl_2_, 0.038 g/L CaCl_2_, 9.8 g/L NaHCO_3_, 4.64 g/L Na_2_HPO_4_ × 2H_2_O, 0.57 g/L KCl, 0.47 g/L NaCl in RO water), 50 drops of defoamer Glanapon DG160 (Bussetti & Co GmbH, Vienna, Austria) and one magnetic stirring bar were added to each bottle. Bottles were closed using septum caps and placed on a multiplex magnetic stirring plate in a water bath heated to 39 °C. The septum of each bottle was penetrated with a water-filled fermentation tube that did not touch the liquid in the bottle. The magnetic stirring plate was set to 300 rpm. Reactors were incubated for 55 min to homogenize the reactor content. After 55 min of incubation (time point 0 h), a zearalenone hydrolase ZenA preparation (ZEN*zyme*^®^; EC 3.1.1.-; BIOMIN Holding GmbH, Getzersdorf, Austria) corresponding to 100 enzyme units (U)/kg feed was added to 4 of the reactors. 1 U of ZenA was defined as the enzyme activity that hydrolyzes 1 μmol ZEN per minute from 15.71 µM ZEN in Teorell Stenhagen buffer [51] adjusted to pH 7.5 containing 0.1 mg/mL bovine serum albumin at 37 °C. The remaining 4 reactors served as control not treated with ZenA. After time point 0 h, reactors were incubated for 3 h. Samples (6 mL) of the fermentation broth were taken using a pipette (tips were covered with filter hoses made from polyester with 105 µm pore size) 10 min, 20 min, 30 min, 1 h, 2 h and 3 h after time point 0 h. The pH was measured in the samples using a handheld pH meter (device 202710, JUMO Mess- und Regelgeräte GmbH, Vienna, Austria). The pH was 6.6 at the start of the experiment and dropped to 6.2–6.3 after 3 h of incubation. For analysis of ZEN and its metabolites, 0.5 mL of each sample was mixed with 1 mL acetonitrile in a 1.5 mL reaction tube. The mixture was vortexed (1 min at maximum speed) and subsequently centrifuged (9 min at 16,600× *g*). The supernatant was transferred to a new 1.5 mL reaction tube and stored at −20 °C.

#### 5.1.1. Analysis of ZEN and Its Metabolites

Acetonitrile (LC-MS grade) was purchased from ChemLab (Zedelgem, Belgium). Acetic acid (99.8–100.5%, AnalaR NORMAPUR) was purchased from VWR International GmbH (Vienna, Austria). Ultrapure water (conductivity ≤ 0.056 µS/cm at 25 °C) was produced using a Sartorius water purification system (Sartorius, Göttingen, Germany). ZEN, α-ZEL and β-ZEL Biopure reference standards were purchased from Romer Labs (Tulln, Austria). HZEN and DHZEN standards (purity > 95%) were produced as described by Fruhauf et al. [21]. Samples were analyzed on a 1290 Infinity HPLC system (Agilent Technologies, Waldbronn, Germany) coupled to a 5500 QTrap mass spectrometer equipped with an electrospray ionization (ESI) source (SCIEX, Foster City, CA, USA). Chromatographic separation was achieved on a Kinetex EVO-C18 column (2.6 µm, 150 × 2.1 mm, 110 Å; Phenomenex, Torrance, CA, USA) and EVO-C18 pre-column (flow rate: 0.35 mL/min; column temperature: 30 °C; duration: 9.5 min; injection volume: 2 µL). Mobile phase A and B consisted of acetonitrile:water:acetic acid (A = 5:94.9:0.1, vol/vol/vol; B = 95:4.9:0.1, vol/vol/vol). The gradient started at 25% B which was held for 0.5 min. Subsequently, the proportion of B was increased to 60% from 0.5 to 3.2 min, increased to 68% from 3.2 to 6.0 min and increased to 100% from 6.0 to 6.1 min. The proportion of B was kept at 100% from 6.1 until 7.6 min, subsequently decreased to 25% from 7.6 to 7.7 min and finally kept at 25% from 7.7 until 9.5 min. Mass spectrometric detection was carried out in multiple reaction monitoring mode with negative polarity (Table 2). LODs for ZEN, α-ZEL, β-ZEL, HZEN and DHZEN were 2.83 nmol/L (0.9 µg/L), 2.81 nmol/L (0.9 µg/L), 2.81 nmol/L (0.9 µg/L), 2.68 nmol/L (0.9 µg/L) and 10.27 nmol/L (3 µg/L), respectively. Limits of quantification (LOQs) for ZEN, α-ZEL, β-ZEL, HZEN and DHZEN were 9.43 nmol/L (3 µg/L), 9.37 nmol/L (3 µg/L), 9.37 nmol/L (3 µg/L), 8.93 nmol/L (3 µg/L) and 30.81 nmol/L (9 µg/L), respectively. Recovery of ZEN, α-ZEL, β-ZEL, HZEN and DHZEN after extraction is described in Table 3.

#### 5.1.2. Statistical Analysis (In Vitro Trial)

Statistical analysis was performed using R software (R Core Team, R Foundation for Statistical Computing, Vienna, Austria) version 3.6.3 using the additional packages readxl version 1.3.1 for reading in raw data, tidyverse version 1.3.0 for data handling and pastecs version 1.3.21 for concise descriptive statistics. Concentrations of ZEN and ZEN metabolites were compared between treatments using one-sided Mann Whitney tests. One-sided tests were used as the enzyme has a directional effect on ZEN and its metabolites (decrease of ZEN and ZEN metabolites α-ZEL and β-ZEL; increase of HZEN and DHZEN). If a compound was above the LOD but below the LOQ, the concentration in the respective sample was assumed to be LOQ/2 for statistical analysis. If a compound was below the LOD, the concentration in the respective sample was assumed to be LOD/2 for statistical analysis. The level of significance was specified to be *p* < 0.05.

### 5.2. Feeding Trial in Rumen-Fistulated Dairy Cows

#### 5.2.1. Experimental Setup

Ruminal metabolism of ZEN with and without supplementation of ZenA was investigated in four rumen-fistulated Holstein Friesian cows (sex: female; age at start: 5.9 ± 1.0 years; body weight at start: 863 ± 65 kg; physiological stage: non-lactating; general health: good, non-gestating; identification procedure: ear tag 4-digit code). The cows were part of the dairy herd of VetFarm (Teaching and Research Farm) of the University of Veterinary Medicine Vienna in Kremesberg, Austria. All procedures related to these experiments were performed according to Austrian law and following the European Guidelines for the Care and Use of Animals for Research Purpose [52]. The animal experiment was approved by ethics and animal welfare commission of the University of Veterinary Medicine, Vienna and the Austrian Federal Ministry of Education, Science and Research (68.205/0156-WF/V/3b/2017, 30.08.2017).

Animals were housed in a separate pen of a loose-housing stable equipped with resting pens covered with straw. Conditions corresponded to good practice in dairy farms, with sufficient airflow and natural light/dark cycles. Animals had free access to roughage feed (Table 4 and Table 5) via computer-regulated access gates (Insentec B.V., Marknesse, The Netherlands). They furthermore had free access to drinking water and mineral licking stones. Qualified veterinary personnel examined the general clinical status of the cows daily. Monitoring included body temperature, ruminal movement and pulse and chewing activity. No medication was administered.

In our trial setup, each animal was considered an experimental unit (*n* = 4). Each animal received the same treatment and served as its own control. First, each animal received ZEN-contaminated concentrate feed. Following a 48-h washout period, each animal received ZEN-contaminated concentrate feed supplemented with ZenA. Details of the experimental setup are shown in Table 6. In brief, for acclimatization, on the day prior to the start of the feeding trial, each animal was manually fed 4 × 500 g of control concentrate (Table 4 and Table 5). Thereafter, animals were manually fed (i) 1 × 500 g concentrate contaminated with ZEN and 3 × 500 g control concentrate on experimental day 1 (ZEN treatment), (ii) 4 × 500 g control concentrate on day 2 (ZEN washout), (iii) 1 × 500 g ZEN-contaminated concentrate supplemented with ZenA and 3 × 500 g control concentrate on day 3 (ZEN+ZenA treatment), and (iv) 4 × 500 g control concentrate on day 4 (ZEN+ZenA washout; Table 6). Animals were treated and assessed in the same random order at each experimental time point.

Dietary ZEN exposure of cows in this trial complied with the guidance value for ZEN in feedingstuff for dairy cows that is in effect in the European Union (EU). The EU guidance value for ZEN in feedingstuff for dairy cows is 0.5 mg/kg relative to feed with a moisture content of 12% [53]. On experimental days 1 and 3, each cow consumed on average 11.27 kg dry matter per day and received a one-time dose of 5 mg ZEN per day (Table 6). Therefore, each cow received on average 0.44 mg ZEN per kg dry matter, or 0.39 mg ZEN per kg feed assuming that the feed had a moisture content of 12%.

The investigated outcome of this trial was the kinetics of ZEN and its metabolites α-ZEL, β-ZEL, HZEN and DHZEN in three reticulorumen locations and in feces following application of ZEN-contaminated concentrate feed with and without supplementation of ZEN-degrading enzyme ZenA.

#### 5.2.2. Preparation of ZEN-Contaminated and ZenA-Supplemented Feed

For production of ZEN-contaminated concentrate feed, lyophilizate of ZEN with maltodextrin as a carrier was prepared. To this end, in three parallel preparations, 50 mg ZEN (Fermentek, Jerusalem, Israel) was dissolved in 1 L RO water (pH 12; adjusted with NaOH) using a magnetic stirrer set to 400 rpm and 37 °C for 20 min. Subsequently, 100 g maltodextrin DE19 (Agrana, Vienna, Austria) was added and dissolved by stirring (400 rpm) at room temperature for 10 min. The solution was lyophilized. Lyophilizate was mixed into concentrate feed at an inclusion rate of 2%. The final concentration of ZEN in feed was aimed to be 10 mg/kg and was verified using HPLC-MS/MS analysis as described below. For production of ZEN-contaminated, ZenA-supplemented concentrate, a zearalenone hydrolase ZenA preparation (ZEN*zyme*^®^; EC 3.1.1.-; BIOMIN Holding GmbH, Getzersdorf, Austria) was mixed into ZEN-contaminated concentrate to achieve an activity of 128 U/kg feed. Same as in the in vitro trial, 1 U of ZenA was defined as the enzyme activity that hydrolyzes 1 μmol ZEN per minute from 15.71 µM ZEN in Teorell Stenhagen buffer [51] adjusted to pH 7.5 containing 0.1 mg/mL bovine serum albumin at 37 °C.

#### 5.2.3. Analysis of Mycotoxin Concentrations in Feed

The presence of the most relevant mycotoxins (ZEN, aflatoxins, trichothecenes, fumonisins, ochratoxin A) in control concentrate, hay and grass silage was determined by Romer Labs GmbH (Tulln, Austria) using HPLC-MS/MS analysis. In case of hay and grass silage, concentrations of all mycotoxins were below the LOQ. In the control concentrate, deoxynivalenol (423 µg/kg), ZEN (104 µg/kg) and fumonisins (fumonisins B1+B2: 355 µg/kg) were found to be naturally present.

For analysis of ZEN concentrations in ZEN-contaminated concentrate and ZEN-contaminated, ZenA-supplemented concentrate, ZEN was extracted by incubating 1 g of feed in 15 mL 80% acetonitrile on a rotary shaker at room temperature for 30 min. The sample was centrifuged at 3500 rpm for 10 min and the supernatant was transferred to a clean 50 mL reaction tube. The feed sample was again incubated with 15 mL 80% acetonitrile on a rotary shaker at room temperature for 30 min. After centrifugation (10 min at 3500 rpm), the supernatant was removed and combined with the previously produced supernatant. A 1 mL aliquot of the pooled supernatant was again centrifuged for 5 min at 3500 rpm. The resulting supernatant was transferred to HPLC vials and stored at −20 °C. ZEN was analyzed using HPLC-MS/MS as described below (see subsection “Analysis of ZEN and its Metabolites in Rumen Fluid and Feces”).

#### 5.2.4. Sampling

On days 1 and 3 of the trial (ZEN and ZEN+ZenA treatment, respectively), ruminal digesta samples were taken from reticulum, ventral sac and dorsal mat layer (Figure 3) immediately before concentrate feeding (0 h), as well as 15 min, 1 h, 2 h, 4 h, 7 h and 10 h after concentrate feeding (Table 6). On days 2 and 4 of the trial (washout days), ruminal digesta samples were taken immediately before first concentrate feeding (0 h), as well as 10 h after first concentrate feeding (Table 6). Ruminal digesta samples (10 mL) were taken via a rumen fistula from reticulum and ventral sac (10 cm above the bottom of the rumen) using a pump as described previously [54]. Samples were taken from the dorsal mat layer 20 cm below the gas phase and rumen fluid was obtained by manually squeezing the samples as described previously [54]. Samples were mixed 1:2 with acetonitrile by vortexing. Subsequently, samples were centrifuged (10 min, 19,000× *g*). Supernatants were transferred to a clean reaction tube and stored at −20 °C.

On days 1 and 3 of the experiment, feces samples were taken from the rectum immediately before concentrate feeding (0 h), as well as 10 h after concentrate feeding (Table 6) and stored at −20 °C. Aliquots (500 mg) of thoroughly homogenized feces samples were extracted for HPLC-MS/MS analysis according to the method described by Binder and coworkers [55].

#### 5.2.5. Analysis of ZEN and Its Metabolites in Rumen Fluid and Feces

HPLC-MS/MS analysis of ZEN, α-ZEL, β-ZEL, HZEN and DHZEN in rumen fluid and feces was performed as described for the in vitro rumen fermentation experiment (see section “In Vitro Rumen Fermentation Experiment”, subsection “Analysis of ZEN and its Metabolites”), with the following modifications. Chromatographic separation was performed with a flow rate of 0.6 mL/min, a duration of 4 min and an injection volume was 1 µL. The gradient started at 30% B which was held for 0.3 min. Subsequently, the proportion of B was increased to 80% from 0.3 to 2 min, and increased to 100% from 2 to 2.1 min. The proportion of B was kept at 100% from 2.1 to 3 min, subsequently decreased to 30% from 3 to 3.1 min and finally kept at 30% from 3.1 until 4 min. LODs for ZEN, α-ZEL, β-ZEL, HZEN and DHZEN in rumen fluid were 2.83 nmol/L (0.9 µg/L), 2.81 nmol/L (0.9 µg/L), 2.81 nmol/L (0.9 µg/L), 2.68 nmol/L (0.9 µg/L) and 10.27 nmol/L (3 µg/L), respectively. LOQs for ZEN, α-ZEL, β-ZEL, HZEN and DHZEN in rumen fluid were 9.43 nmol/L (3 µg/L), 9.37 nmol/L (3 µg/L), 9.37 nmol/L (3 µg/L), 8.93 nmol/L (3 µg/L) and 30.81 nmol/L (9 µg/L), respectively. LODs for ZEN, α-ZEL, β-ZEL, HZEN and DHZEN in feces were 0.03 nmol/g (8.4 ng/g), 0.03 nmol/g (8.4 ng/g), 0.03 nmol/g (8.4 ng/g), 0.03 nmol/g (8.4 ng/g) and 0.10 nmol/g (28 ng/g), respectively. LOQs for ZEN, α-ZEL, β-ZEL, HZEN and DHZEN in feces were 0.09 nmol/g (28 ng/g), 0.09 nmol/g (28 ng/g), 0.09 nmol/g (28 ng/g), 0.08 nmol/g (28 ng/g) and 0.29 nmol/g (84 ng/g), respectively.

#### 5.2.6. Statistical Analysis (Feeding Trial)

Statistical analysis was performed using R software (R Core Team, R Foundation for Statistical Computing, Vienna, Austria) version 3.4.3 using the additional packages readxl version 1.0.0 for reading in rawdata, tidyverse version 1.2.1 for data handling and pastecs version 1.3.21 for concise descriptive statistics. Concentrations of ZEN and ZEN metabolites were compared between treatments using one-sided, paired, non-parametric Wilcoxon Signed Rank tests. One-sided tests were used as the enzyme has a directional effect on ZEN and its metabolites (decrease of ZEN and ZEN metabolites α-ZEL and β-ZEL; increase of HZEN and DHZEN). Paired tests were used as the same animals were used for both treatments. If a compound was above the LOD but below the LOQ, the concentration in the respective sample was assumed to be LOQ/2 for statistical analysis. If a compound was below the LOD, the concentration in the respective sample was assumed to be LOD/2 for statistical analysis. The level of significance was specified to be *p* < 0.05.

Since this was the first study in ruminants of its kind, a priori calculation of statistical power was difficult to conduct. However, previous trials with other farm animals have shown that HZEN is typically not present (<LOD) in relevant matrices from animals receiving ZEN-contaminated feed without ZenA supplementation, while it is usually detectable upon administration of ZenA. Furthermore, the effect of ZenA is directional, so only one-sided tests are required (see above). In a paired, non-parametric test, this means that four animals would reasonably be enough to reject the null hypothesis if this was true (80% statistical power).

## Figures and Tables

**Table 1:** Percentage of ZEN and ZEN metabolite concentrations relative to the sum of these concentrations in reticulorumen and feces.

	ZEN [%] ^1^	α-ZEL [%] ^1^	β-ZEL [%] ^1^	HZEN [%] ^1^	DHZEN [%] ^1^
**Reticulorumen ^2^ ZEN treatment**					
15 min	100.0	0.0 (traces)	0.0 (traces)	0.0	0.0
1 h	84.8	7.6	7.6	0.0	0.0
4 h	84.1	15.9	0.0 (traces)	0.0	0.0
7 h	78.7	21.3	0.0 (traces)	0.0	0.0
10 h	74.4	25.6	0.0	0.0	0.0
24 h	50.2	49.8	0.0	0.0	0.0
**Reticulorumen ^2^ ZEN+ZenA treatment**					
15 min	5.9	0.0 (traces)	0.0	94.1	0.0
1 h	0.0 (traces)	0.0	0.0	100.0	0.0
4 h	0.0 (traces)	0.0	0.0	100.0	0.0 (traces)
7 h	0.0 (traces)	0.0	0.0	100.0	0.0 (traces)
10 h	0.0 (traces)	0.0 (traces)	0.0	100.0	0.0 (traces)
**Feces ^3^ ZEN treatment**					
10 h	11.0	10.9	78.1	0.0	0.0
**Feces ^3^ ZEN+ZenA treatment**					
0 h	15.6	15.5	69.0	0.0	0.0
10 h	0.0 (traces)	9.0	59.1	31.9	0.0

^1^ Abbreviations: ZEN—zearalenone; α-ZEL—α-zearalenol; β-ZEL—β-zearalenol; HZEN—hydrolyzed ZEN; DHZEN—decarboxylated HZEN. ^2^ Percentage calculated based on median of concentrations detected in dorsal mat layer (*n* = 4), ventral sac (*n* = 4) and reticulum (*n* = 4). If a compound was detectable but below the limit of quantification (LOQ), the concentration in the respective sample was assumed to be LOQ/2 for calculation of median. “0.0 (traces)” indicates that the median was 0, but traces of the respective compound (limit of detection < concentration < LOQ) were detected in <50% of the samples. ^3^ Percentage calculated based on median of concentrations detected in feces (*n* = 4). If a compound was detectable but below the LOQ, the concentration in the respective sample was assumed to be LOQ/2 for calculation of median. “0.0 (traces)” indicates that the median was 0, but traces of the respective compound (limit of detection < concentration < LOQ) were detected in <50% of the samples.

**Table 2:** Mass transitions and MS parameters.

Analyte ^1^	Q1 Mass (Da)	Q3 Mass (Da) ^2^	Declustering Potential (V)	Entrance Potential (V)	Collision Energy (V) ^2^	Collision Cell Exit Potential (V) ^2^
ZEN	317.1	131.0/175.0	−100	−10	−42/−34	−9/−7
α-ZEL	319.1	275.1/160.0	−105	−10	−30/−42	−7/−9
β-ZEL	319.1	275.1/160.0	−105	−10	−30/−42	−7/−9
HZEN	335.0	149.0/161.0	−80	−10	−34/−34	−1/−9
DHZEN	291.1	149.0/161.0	−80	−10	−25/−25	−8/−8

^1^ Abbreviations: ZEN-zearalenone; α-ZEL-α-zearalenol; β-ZEL-β-zearalenol; HZEN-hydrolyzed zearalenone; DHZEN-decarboxylated HZEN. ^2^ Quant/qual.

**Table 3:** Recoveries of ZEN and its metabolites after extraction from rumen fluid.

Analyte ^1^	Spike Concentration (µg/L)	Recovery (% of Spike Concentration)
ZEN	3	81
	30	91
α-ZEL	3	105
	30	85
β-ZEL	6	69
	30	78
HZEN	3	82
	30	103
DHZEN	3	65
	30	80

^1^ Abbreviations: ZEN-zearalenone; α-ZEL-α-zearalenol; β-ZEL-β-zearalenol; HZEN-hydrolyzed zearalenone; DHZEN-decarboxylated HZEN.

**Table 4:** Diet ingredients.

**Roughage (% of Dry Matter)**	
Grass silage	50.0
Hay	50.0
**Concentrate (Control; % of Dry Matter)**	
Corn	50.0
Wheat	33.3
Barley grain	8.3
Mineral-vitamin premix ^1^	5.0
CaCO_3_	3.3

^1^ Rindavit TMR 11 Ass-Co Schaumann GmbH & Co KG, Brunn am Gebirge, Austria. Contained 16 g/100 g Ca, 4 g/100 g P, 7 g/100 g Mg, 10 g/100 g Na, 3325 mg/kg Mn, 5000 mg/kg Zn, 1000 mg/kg Cu, 100,000 IU/kg Vitamin A, 1,000,000 IU/kg Vitamin D3 and 4000 mg/kg Vitamin E.

**Table 5:** Nutrient composition.

	Roughage	Concentrate (Control)
Dry matter (DM; % in fresh matter)	54.6	89.6
Ash (% of DM)	7.5	7.9
Crude Protein (% of DM)	13.2	11.0
Ether extract (% of DM)	1.7	2.7
Neutral detergent fiber (% of DM)	57.5	13.7
Acid detergent fiber (% of DM)	37.3	4.3
Non fiber carbohydrates (NFC) ^1^	20.0	64.4

^1^ NFC = 100%−(neutral detergent fiber % + crude protein % + ether extract % + ash %).

**Table 6:** Feeding and sampling schedule.

Day	Treatment	Diet ^1^	Sampling
1	ZEN	Roughage provided ad libitum. Manual feeding of 500 g ZEN-contaminated (10 mg/kg) ^3^ concentrate at 08:30 a.m. Manual feeding of 500 g control concentrate at 11:30 a.m., 2:30 p.m. and 5:30 p.m., respectively.	Rumen fluid: ^2^ immediately before ZEN-contaminated concentrate feeding (0 h), 6 sampling time points 15 min–10 h after ZEN-contaminated concentrate feeding (15 min, 1 h, 2 h, 4 h, 7 h, 10 h) Feces: immediately before ZEN-contaminated concentrate feeding (0 h), 10 h after ZEN-contaminated concentrate feeding (10 h)
2	No treatment (ZEN washout)	Roughage provided ad libitum. Manual feeding of 500 g control concentrate at 08:30 a.m., 11:30 a.m., 2:30 p.m. and 5:30 p.m., respectively.	Rumen fluid: ^2^ immediately before first concentrate feeding (24 h), 10 h after first concentrate feeding (34 h)
3	ZEN+ZenA	Roughage provided ad libitum. Manual feeding of 500 g ZEN-contaminated (10 mg/kg) ^4^ concentrate supplemented with ZenA (128 U/kg) at 08:30 a.m. Manual feeding of 500 g control concentrate at 11:30 a.m., 2:30 p.m. and 5:30 p.m., respectively.	Rumen fluid: ^2^ immediately before ZEN+ZenA concentrate feeding (0 h), 6 sampling time points 15 min–10 h after ZEN+ZenA concentrate feeding (15 min, 1 h, 2 h, 4 h, 7 h, 10 h) Feces: immediately before ZEN+ZenA concentrate feeding (0 h), 10 h after ZEN+ZenA concentrate feeding (10 h)
4	No treatment (ZEN+ZenA washout)	Roughage provided ad libitum. Manual feeding of 500 g control concentrate at 08:30 a.m., 11:30 a.m., 2:30 p.m. and 5:30 p.m., respectively.	Rumen fluid: ^2^ immediately before first concentrate feeding (24 h), 10 h after first concentrate feeding (34 h)

^1^ Composition of roughage and concentrate see Table 4 and Table 5. ^2^ Rumen fluid samples were collected at three locations in the reticulorumen, i.e., ventral sac, reticulum and dorsal mat layer. ^3^ Concentration was verified using HPLC-MS/MS analysis and determined to be 10.7 mg/kg (standard deviation = 1.3 mg/kg). In total, each cow received 5 mg ZEN. ^4^ Concentration was verified using HPLC-MS/MS analysis determined to be 11.4 mg/kg (standard deviation = 0.8 mg/kg). In total, each cow received 5 mg ZEN.

## Data Availability

Data are contained within the article.
